# Anabaenopeptins from *Nostoc edaphicum* CCNP1411

**DOI:** 10.3390/ijerph191912346

**Published:** 2022-09-28

**Authors:** Robert Konkel, Michał Grabski, Marta Cegłowska, Ewa Wieczerzak, Grzegorz Węgrzyn, Hanna Mazur-Marzec

**Affiliations:** 1 Division of Marine Biotechnology, Institute of Oceanography, University of Gdańsk, M. J. Piłsudskiego 46, PL-81378 Gdynia, Poland; 2 Department of Molecular Biology, University of Gdansk, Wita Stwosza 59, PL-80308 Gdańsk, Poland; 3 Institute of Oceanology, Polish Academy of Sciences, Powstańców Warszawy 55, PL-81712 Sopot, Poland; 4 Department of Biomedical Chemistry, Faculty of Chemistry, University of Gdańsk, Wita Stwosza 63, PL-80308 Gdańsk, Poland

**Keywords:** anabaenopeptins, cyanobacteria, *apt* gene cluster, nonribosomal peptide synthetase, biological activity

## Abstract

Cyanobacteria of the *Nostoc* genus belong to the most prolific sources of bioactive metabolites. In our previous study on *Nostoc edaphicum* strain CCNP1411, the occurrence of cyanopeptolins and nostocyclopeptides was documented. In the current work, the production of anabaenopeptins (APs) by the strain was studied using genetic and chemical methods. Compatibility between the analysis of the *apt* gene cluster and the structure of the identified APs was found. Three of the APs, including two new variants, were isolated as pure compounds and tested against four serine proteases and carboxypeptidase A (CPA). The in vitro enzymatic assays showed a typical activity of this class of cyanopeptides, i.e., the most pronounced effects were observed in the case of CPA. The activity of the detected compounds against important metabolic enzymes confirms the pharmaceutical potential of anabaenopeptins.

## 1. Introduction

Anabaenopeptins (APs) are commonly occurring cyanobacteria metabolites assembled by nonribosomal peptide synthetase (NRPS) [1,2]. The peptides were identified for the first time in *Anabaena flos aquae* NRC-525-17 from the Saskatchewan River in Canada and named after the source organism [3]. The production of APs was also documented from *Nostoc* [4,5,6], *Brasilonema* [7,8], *Desmonostoc* [7], *Aphanizomenon* [9], *Anabaena/Dolichospermum* [10,11,12,13,14], *Nodularia* [6,15,16,17,18] (Nostocales order), *Lyngbya/Limnoraphis* [19,20], *Oscillatoria* [21,22,23], *Planktothrix* [24,25,26,27,28,29,30,31], *Tychonema* [32] (Oscillatoriales order), *Microcystis* [11,28,29,33,34,35,36,37,38,39,40] (Chrocococales order), *Woronichinia* [41,42,43] (Synechococales order) and *Schizothrix* [44] (Pseudanabaenales order). Additionally, anabaenopeptin-like compounds, keramamides [45,46] and konbamide [47], were found in marine sponges *Theonella* and *Melophlus* (*Psammocinia* aff. *bulbosa*) [45,46,47,48,49]. As the AP gene cluster was also detected in the tectomicrobial bacterium, the potential for the production of the peptides by other bacteria was suggested [4].

The structure of APs comprises a five-membered peptide ring linked through the ureido bond with one amino acid side chain. The general formula of this class of peptides is X^1^-CO-[Lys^2^-X^3^-X^4^-MeX^5^-X^6^], where Lys^2^ in D-configuration is the only conservative amino acid in the structure [35,50,51]. Positions 4 and 5 are often occupied by homo-amino acids (e.g., Hph, Hty) and *N*-methylated amino acids (e.g., MeAla, MeHty, MeAsn or MeHph).

The structural diversity of APs is determined by the organisation of the gene cluster and the corresponding modular NRPS multienzyme complex. Each module catalyses the activation and incorporation of an amino acid into a peptide chain. The modules contain several catalytic domains that are responsible for the subsequent steps of the process. These include the adenylation domain (A), responsible for the recognition and activation of a specific amino acid residue, the thiolation domain (T) that transfers the peptide between the domains, the condensation domain (C) that catalyses the formation of the peptide bond, and the thioesterase domain (Te) that is a part of the last module. Te is responsible for the release and, optionally, for the cyclisation of the free peptide [4,6]. Anabaenopeptin NRPS also contains the epimerase domain (E) in the Lys^2^ activating module and the methyltransferase domain that catalyses *N*-methylation of residues at position 5 [4,6]. The biosynthesis of the peptides proceeds according to the collinearity rule so that the number of modules corresponds to the number of residues in the peptide chain.

Peptides with typical AP structural features were named arbitrarily, so their nomenclature is not systematic. Among the 155 identified APs [51], there are structural variants of the compounds named nodulapeptins [15], brunsvicamides [32], ferintoic acids [52], lyngbyaureidamides [20], nostamides [4,6], oscillamides [21,24], pompanopeptins [19], schizopeptin [44], mozamides [48], paltolides [53] and psymbamide [54]. Of these, 38 are produced by cyanobacteria of the genus *Nostoc*, isolated mainly from terrestrial environments (Table 1 and Table S1).

**Figure 1:**
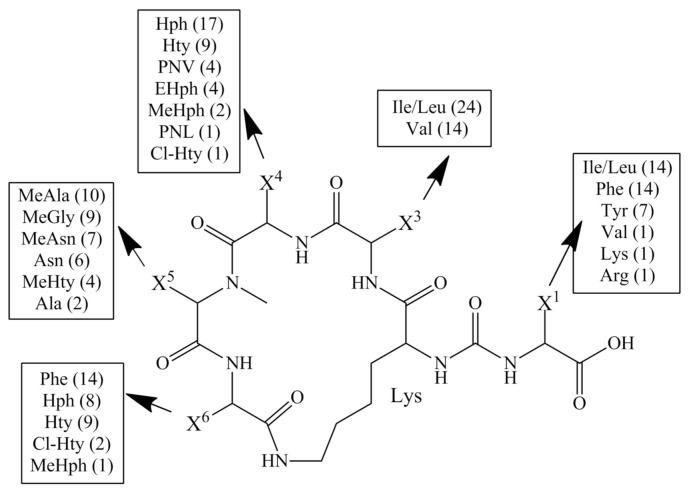
General structure of anabaenopeptins detected in cyanobacteria of the *Nostoc* genus. The number of variants with specific amino acids is given in the brackets.

In the peptides produced by *Nostoc*, amino acid residues common for other AP variants are present, e.g., in the exocyclic position, Ile/Leu, Phe, Val, Lys or Arg can be found, while position 3 is occupied by Ile/Leu or Val and aromatic amino acids are in position 6 (Figure 1). The unique feature of APs from *Nostoc* is phenylnorvaline (PNV) and phenylnorleucyne (PNL) in position 4 [56] and the presence of Cl-substituted Hty in positions 4 and 6 [56]. The structural diversity of APs results in a wide range of activities revealed in the in vitro assays. The peptides inhibit protein phosphatases [24,57], elastase [40,58,59], carboxypeptidase A (CPA) [9,23,57], carboxypeptidase B [60] and TAFIa (activated thrombin-activable fibrinolysis inhibitor) [49,56,61].

In our previous work, sequencing of the *Nostoc edaphicum* CCNP1411 genome revealed the presence of a region similar to the anabaenopeptin synthetase gene cluster. In the current work, the organisation of the gene cluster was studied and compared with the results of structural analysis of the detected APs performed with the application of LC-MS/MS and NMR. In addition, three of the APs were isolated from the collected biomass and tested against four serine proteases and carboxypeptidase A.

## 2. Materials and Methods

### 2.1. NRPS Alignment

The alignment of anabaenopeptin synthetase gene clusters (GenBank numbers GU174493 and HM773422) [1,6] to the selected region was carried out with BLASTn [62]. Correction of the gene prediction was achieved using Prodigal [63]. Genes found within the aligned regions were subjected to the NCBI Conserved Domain Database search (CDD v3.19) to determine the evolutionary conserved protein domains and motifs [64]. The recognition of the residue positions of amino acids in the substrate-binding pocket of adenylation domains was performed manually, according to Stachelhaus et al. [65]. The CGView Comparison Tool [66] was used to create a map of the genome fragment.

### 2.2. Extraction and Isolation of Anabaenopeptins

*Nostoc edaphicum* CCNP1411 was isolated from the Gulf of Gdańsk and grown for biomass as previously described [67]. The lyophilised biomass of *N. edaphicum* CCNP1411 (80 g) was homogenised with a mortar and pestle and extracted twice with 75% methanol (MeOH) in MilliQ water (2 × 500 mL) by vortexing for 15 min. The combined extracts were centrifuged (10,000× *g*; 15 min; 4 °C) and diluted to a MeOH concentration lower than 10%. To assess the relative content of the AP variants in *N. edaphicum* CCNP1411, the cells (23 mg) were additionally extracted twice with 75% MeOH (2 × 5 mL) by 15 min vortexing. The supernatants were evaporated to dry residue and redissolved in 75% MeOH (1 mL).

The separation of the compounds was performed with flash and preparative chromatography using the Shimadzu HPLC system (Shimadzu Corporation, Kyoto, Japan). First, the extract was loaded into a 120 g SNAP cartridge KP-C18-HS (Biotage, Uppsala, Sweden) at a flow rate of 20 mL·min^−1^. The elution started with MilliQ water, and every 17.5 min, the content of MeOH increased by 10% until it reached 100% MeOH. The 40-L fractions were collected and analysed with an LC-MS/MS system. The fractions containing anabaenopeptins were combined, evaporated in a vacuum concentrator (MiVac, SP Scientific, Ipswich, UK) and separated in a Jupiter Proteo C_12_ column (250 × 21.2 mm, 4 μm, 90 Å) (Phenomenex, Aschaffenburg, Germany) by repeated preparative chromatography. The mobile phase was composed of a mixture of 5% acetonitrile in MilliQ water (phase A) and 100% acetonitrile (phase B), both with 0.1% formic acid. In the chromatographic runs, gradients from 5% B to 99% B were used. The collected fractions (2 mL each) were analysed with LC-MS/MS.

### 2.3. LC-MS/MS Analysis

The LC-MS/MS system was composed of Agilent 1200 HPLC (Agilent Technologies, Waldbronn, Germany) and a QTRAP5500 tandem mass spectrometer. The compounds were separated in a Zorbax Eclipse XDB-C_18_ column (4.6 × 150 mm, 5 μm) (Agilent Technologies, Santa Clara, CA, USA). Gradient elution (0.6 mL·min^–1^) was performed with the same mobile phases as in the preparative analysis. The turbo ion spray operated at 550 °C; voltage, 5.5 kV; nebuliser gas pressure, 60 psi; curtain gas pressure, 20 psi. To determine the content of the samples, an IDA (information-dependent acquisition) mode was used, and ions within the *m*/*z* range 500–1250 and intensity higher than 5 × 10^5^ cps were fragmented. The assessment of the relative content of APs in the extract was performed in multiple reaction monitoring mode (MRM). The following transitions were monitored: 807→402, 231, 120 for anabaenopeptin AP806Ne (*m*/*z* 807); 821→448, 248, 120 for AP820Ne (*m*/*z* 821); 835→448, 248, 120 for AP SA6 (*m*/*z* 835); and 837→448, 248, 120 for AP836Ne (*m*/*z* 837). The collision energy was 60 eV, and the dwell time was 100 msek.

### 2.4. NMR Analysis

^1^D 1H-NMR and 2D NMR (COSY, TOCSY, and ROESY) were acquired on a Varian Unity Inova 500 spectrometer (500 MHz). Spectra were recorded in DMSO-d_6_. NMR data were processed and analysed with TopSpin (Bruker) and POKY software [68].

### 2.5. Enzymatic Assays

The enzyme inhibitory activity of anabaenaopeptins was assayed against trypsin [69], chymotrypsin [70], thrombin [70], elastase [71] and carboxypeptidase A [70]. The samples were serially diluted (1 mg, 1:1–1:10,000 times) in 1% DMSO; the standard inhibitors were also prepared in 1% DMSO (Table S2). The mixtures containing the anabaenopeptins or positive control (standard inhibitors) were preincubated for 5–20 min in a microplate reader (Varioskan Flash Thermo Fisher Scientific OY, Vantaa, Finland) with the addition of the enzyme and buffer (Table S2). As a negative control, 1% DMSO, without the addition of the enzyme, was used. Then, the substrates were added (Table S2), and mixtures were incubated for an additional 10 or 20 min (Table S2). The absorbance was measured at 350 nm (carboxypeptidase A) or at 405 nm (other enzymes). The tests were performed in triplicates.

## 3. Results

### 3.1. Anabaenopeptin Nonribosomal Peptide Synthetase (NRPS) Gene Cluster

**Figure 2:**
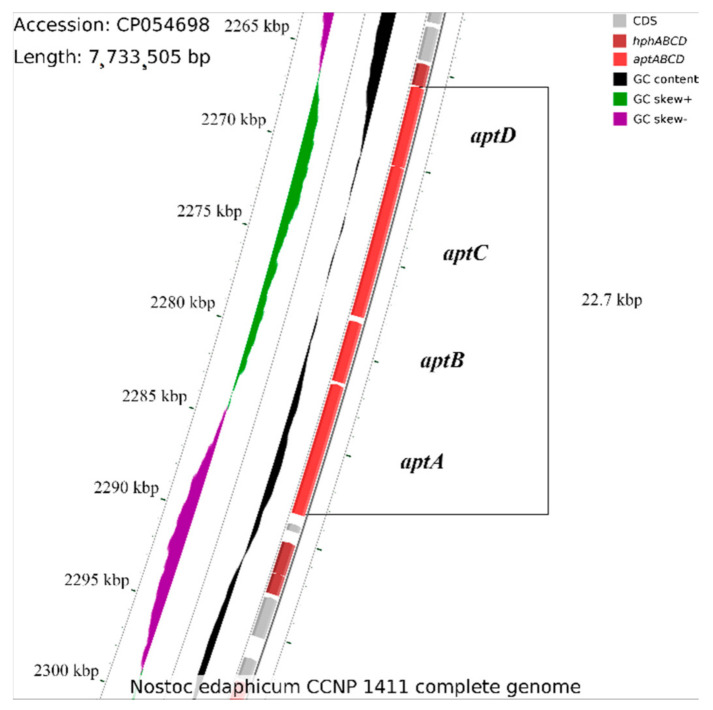
Fragment of the *Nostoc edaphicum* CCNP1411 chromosome, encoding the anabaenopeptin synthetase gene cluster (red).

Although the anabaenopeptin synthetase gene cluster has never been studied in *N. edaphicum* CCNP1411, it was suggested that nonribosomal anabaenopeptin synthetase might be encoded within its genome [65]. Nevertheless, given spans were inaccurately indicated, in which only a fragment of the gene coding for potential synthetase overlaps with some extended spans of the studied gene cluster. The core structure of the potential anabaenopeptin synthetase cluster is located between the 2,265,881 and 2,288,626 positions within the *N. edaphicum* CCNP1411 chromosome, and it consists of four genes (locus tags HUN01_12140, HUN01_12145, HUN01_12150 and HUN01_12160) (Figure 2, Table 2). Identified genes are described as those hypothetically encoding proteins being amino acid adenylation domain-containing proteins. The cluster was found on the complementary strand; therefore, locus tag numbers are in the descending order.

The first NRPS ORF, *apt*A (HUN01_12160), is a 6957 bp long gene, coding for a protein comprised of two modules, one containing the adenylation domain (A) and the second identified as the peptidyl carrier protein (PCP). The first module, lacking the condensation (C) domain but containing the adenylation domain, was predicted (based on the nonribosomal consensus code [65,72]) to be involved in the activation of Leu, Ile or Val, which become amino acid substrates (Table 3). PCP is the shuttle to the nascent (C) catalytic domain of the second module. No mutations were found in the region encoding the HHXXXDG motif of this condensation domain or every other condensation domain found within this cluster, suggesting that a peptide bond between nascent peptides forming the peptide chain may occur. The epimerase domain, included in the second module, explains the stereochemistry of Lys (D-Lys), an amino acid activated by the second module adenylation domain, whose amino group was found on the right side in its Fisher projection.

The second ORF, the *apt*B gene (HUN01_12150), is a 3231 bp long DNA locus encoding a polypeptide that contains one module. The condensation domain is similar to the DCL-type protein, which catalyses bond formation between the donor D-Lys and the L-amino acid acceptor, namely Leu activated by the adenylation domain.

The third ORF, *apt*C (HUN01_12145), encodes a protein containing two modules with methyltransferase found nascent to the C-terminus of the second module adenylation domain. The former module was found ambiguous. It appears that the signature sequence of the adenylation domain might activate Phe; nonetheless, such an assessment is based on residue positions obtained from not one but several reference domains. However, in favour of this prediction, the adenylation domain of the *apt*C-encoded module was found to possess Thr and Ile at positions 278 and 299, respectively, which were found exclusively in the Phe-activating reference domains. The amino acid activated by the second adenylation domain is Asn, presumably methylated by the methyltransferase and transferred further by the PCP domain found at the C-terminus of the *apt*C gene product.

The start codon of the fourth ORF, putatively encoding NRPS, was found to overlap the stop codon of *apt*C, suggesting that *apt*CD may be transcribed as an operon. The *apt*D gene (HUN01_12140), composed of 4215 bp, encodes a protein, which, apart from the main module, also contains the *N*-terminal docking domain and the thioesterase domain at the C-terminus, required to release the peptide.

Upstream of the *apt*ABCD synthetase gene cluster, a gene (HUN01_12135) was found, which translated a sequence that was homologous (88% identity) to HphA. As Hph is present in the produced peptide, further investigations revealed that besides HUN01_12135, the genes HUN01_12175 and HUN01_12180 are putatively synonymous with the *hph*CD and *hph*B genes, respectively, which are essential for the homo-amino acid biosynthetic pathway [73]. These two genes are located downstream of the *apt*A gene, and their transcripts were found to be 91% identical to those derived from *Nostoc punctiforme* (GenBank accession number WP_012409012). The core structure of the anabaenopeptin synthetase gene cluster was aligned against three previously characterised hypothetical anabaenopeptin-producing cyanobacterial strains, namely *Nodularia spumigena* CCY9414, *Anabaena* sp. 90 and *Nostoc punctiforme* PCC 73,102 [74]. Although the structures of clusters derived from *Nodularia* and *Anabaena* exhibit overall similarity, as evidenced by the percentage of identities (mean percentage identity = 78.48), the *Nostoc punctiforme* NRPS sequence does not resemble the structure of the anabaenopeptin synthetase gene cluster (Figure S1).

### 3.2. Identification of AP Structures

**Figure 3:**
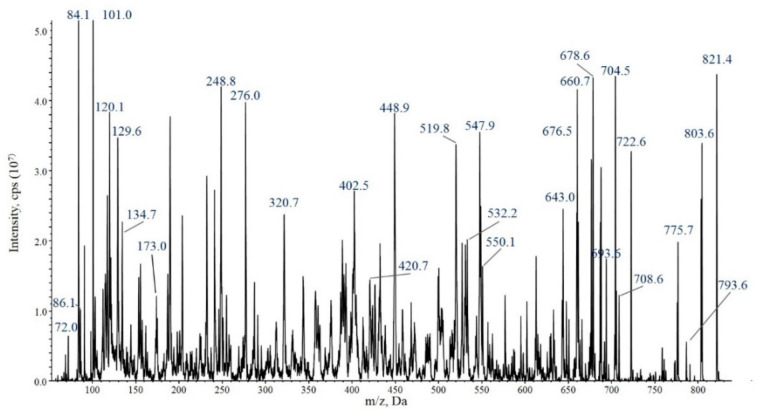
Enhanced product ion mass spectrum of the anabaenopeptin AP820Ne [Lys–Leu–Hph–MeAsn–Phe]CO–Val with precursor ion [M+H]^+^ at *m*/*z* 821. The mass signals were assigned to the following fragments: 821 [M+H]^+^, 803 [M+H–H_2_O]^+^, 793 [M+H–CO]^+^, 775 [M+H–CO–H_2_O]^+^, 722 [M+H–Val]^+^, 708 [M+H–Leu]^+^, 704 [M+H–Val–H_2_O]^+^, 693 [M+H–MeAsn]^+^, 678 [M+H–Val–CO]^+^, 660 [M+H–Hph]^+^, 643 [M+H–Hph–H_2_O]^+^, 550 [M+H–(Lys+CO+Val)]^+^, 547 [M+H–(Leu+Hph)]^+^, 532 [M+H–(Lys+CO+Val)–H_2_O]^+^, 519 [M+H–(Leu+Hph)–CO]^+^, 448 [M+H–Val–(Hph+Leu)]^+^, 420 [M+H–Val–(Hph+Leu)–H_2_O]^+^, 402 [M+H–Val–(Hph+Leu)–H_2_O–CO]^+^, 276 [Phe+MeAsn+H]^+^, 248 [Phe+MeAsn+H–CO]^+^, 173 [Lys+CO+NH_2_+H]^+^, 134 Hph immonium ion, 129 [Lys+2H]^+^, 120 Phe immonium ion, 86 Leu immonium ion, 84 Lys-derived ions^+^ and 72 Val immonium ion.

The nontargeted LC-MS/MS analysis of the *N. edaphicum* CCNP 1411 cell extract in IDA mode did not reveal the presence of any anabaenopeptin variants. Variants of four anabaenopeptins with pseudomolecular ions ([M+H]^+^) at *m*/*z* 807, 821, 835 and 837 (Table 4) were only detected when several chromatographic fractions were combined and concentrated. MRM analyses were performed to determine the relative amount of anabaenopeptins produced by *N. edaphicum*. The peak area of AP820Ne in the MRM chromatogram was the largest (9.63 × 10^5^ cps) compared with AP SA6 (4.49 × 10^4^ cps), AP836Ne (2.19 × 10^4^ cps) and (1.84 × 10^4^ cps) (Figure S2). Structures of the peptides were elucidated based on the analysis of mass fragmentation spectra with some diagnostic ions, including immonium ions and other ions that correspond to specific fragment ions formed during collision-induced dissociation (Figure 3 and Figures S3–S5). Positions 2, 3 and 6 of the peptides were found to be conserved and occupied by Lys^2^, Leu/Ile^3^ and Phe^6^, respectively. The applied genetic and chemical methods (MS/MS and NMR) did not allow for the distinction between Leu and Ile; therefore, in this work, the residue is marked as Leu*. The most significant diagnostic ions were: Lys-derived ions at m/z 84, 101 and 129; immonium ions of Phe (120), Hph (134), MeAsn (101), Leu* (86) and Val (72); ions generated by the cyclic part of APs at *m*/*z* 664, 678, 678 and 694 (for AP807Ne, AP820Ne, AP SA6 and AP836Ne, respectively); and [M+H-X1-(X3+X4)]^+^ ion at *m*/*z* 434 for AP807 and at *m*/*z* 448 for AP820Ne, AP SA6 and AP836Ne. In all MS/MS spectra of MeAsn-containing APs, the [MeAsn+Phe+H-CO]^+^ ion at *m*/*z* 248 is present. Other ions that confirmed the structure of AP are shown in Figure 3 and Figures S3–S5.

**Figure 4:**
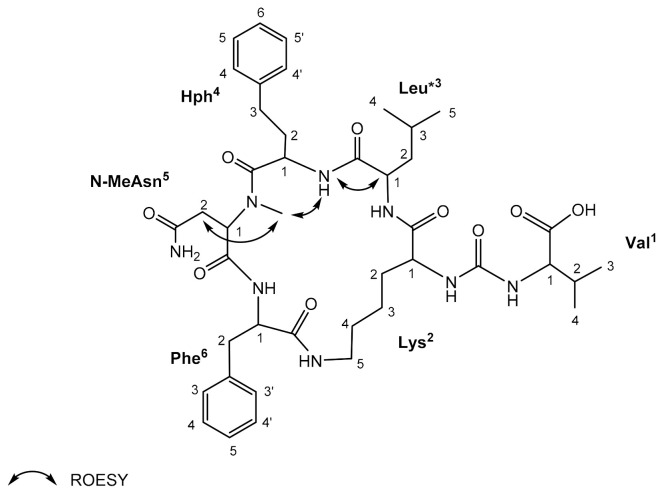
Key ROESY correlations in anabaenopeptin AP820Ne identified in *Nostoc edaphicum* CCNP1411.

For AP820Ne isolated from *N. edaphicum* CCNP1411 in the highest amounts (~1 mg), structural analysis by NMR was possible. The ^1^H-NMR spectra (Figures S6–S9) of the studied compound displayed the typical pattern of a peptide (i.e., amide protons δ_H_ 6.62–9.02 ppm and protons α to carbonyl in amino acids δ_H_ 3.53–5.33 ppm). The COSY and TOCSY experiments allowed to assign NMR spin systems to Val, Lys, Leu*, Hph, *N*-MeAsp and Phe (Figure 4, Table 5). The presence of aromatic amino acid residues was recognised by the signals occurring in the aromatic region of the spectrum (δ_H_ 7.01–7.32 ppm). The sequence assignments based on NMR data corroborated the results of the MS experiments of the compound and are consistent with published data [40].

### 3.3. Enzymatic Assays

Of the four identified APs, only three were isolated as pure compounds and in sufficient amounts to perform enzyme inhibition assays. These were AP SA6, AP820Ne and AP806Ne. In vitro experiments did not reveal the activity of the peptides against trypsin and chymotrypsin, and their activity against thrombin was only observed at the highest concentration used in the assay (45 µg mL^–1^) (Table 6). The three tested APs inhibited the activity of carboxypeptidase A. The IC_50_ value for AP806Ne was higher (21.0 µM) than for AP820Ne (3.53 µM) and AP SA6 (4.5 µM), indicating lower activity of AP806Ne. The latter two APs were also active against elastase and inhibited the enzyme with IC_50_ = 5.5 µM and 22.7 µM, respectively.

## 4. Discussion

In this work, the anabaenopeptin biosynthetic gene cluster and its products were analysed. A genome of a cyanobacterial strain usually contains several NRPS gene clusters, and within one class of the gene products, numerous structural variants are produced. Analysis of 184 cyanobacterial genomes deposited in the NCBI GeneBank showed a positive correlation between genome size (1.65–12.05 Mb) and the number of natural product biosynthetic gene clusters [75]. In line with this rule, cyanobacteria of the Nostocales order (including *Nostoc*) belong to the most prolific sources of natural products [76]. They synthesise numerous bioactive metabolites classified as peptides, lipopeptides, fatty acids, alkaloids and terpenoids [35,77,78,79,80]. In our previous studies, two classes of nonribosomal peptides, cyanopeptolins [81] and nostocyclopeptides [67], were identified in *N. edaphicum* CCNP1411 (total genome size 8.31 Mb, including five plasmids). In addition, a region similar to the anabaenopeptin gene cluster was found in the chromosome [67]. To check *apt* gene expression, LC-MS/MS analysis of *N. edaphicum* extract was performed, but APs were not detected. This result suggested either lack of *apt* gene expression or production of the peptides in trace amounts. The latter option was confirmed when APs were detected in the analyses of concentrated *N. edaphicum* biomass. Of these, AP SA6 is the only AP that has been previously reported [50,56], while the other three are new structural variants. Their structures were found to be characteristic of APs produced by cyanobacteria of the genus *Nostoc*. All positions, especially in the cyclic part of the molecule, are occupied by the residues that were most frequently reported in APs identified in *Nostoc* (Figure 1). The occurrence of MeAsn/Asn in position 5 is quite rare and unique to *Nostoc* [5,7,56] or *Desmonostoc* [7], previously classified as *Nostoc* [82]. To distinguish the new APs from the already known variants with the same molecular masses (and the same *m*/*z* values), we added two letters in their symbols (Ne = *N. edaphicum*). However, even with these symbols, some confusion might occur when new APs are detected in other *N. edaphicum* strains. As has already been postulated by other authors, with the growing number of new anabaenopeptin variants, their nomenclature needs to be systematised.

In different taxonomic groups of cyanobacteria, the organisation of the NRPS gene clusters involved in the biosynthesis of anabaenopeptins is similar. However, even in strains of the same species, the products of the genes can vary. Namely, the specific positions in the AP structure can be occupied by different amino acid residues. The structural diversity of NRPs encoded by a single NRPS results mainly from the promiscuity of A domains responsible for the selection and incorporation of amino acids [1,6]. In *Anabaena* sp. 90, the diversity is additionally increased by the presence of two starter modules with different substrate specificity of A domains activating the first amino acid residue [6]. In *N. edaphicum* CCNP141, the 22.7 kb *apt* gene cluster was found to be similar in size and organisation to the *apt* gene clusters described in other cyanobacteria [1,4,6,83]. The cluster is composed of four genes encoding four NRPS enzymes (*aptABCD*) containing six modules. Moreover, the predicted substrate specificity of A domains in the enzymatic complex catalysing AP synthesis supported the identification of specific amino acids in AP structures performed by MS/MS analyses. The detection of the epimerase domain in the second module corroborates the presence of D-Lys in position 2. In addition, the presence of methyltransferase at the *C*-terminus of the *aptC* gene product and the detection of genes synonymous with the *hphCD* and *hphB* genes confirmed the presence of homo-amino acid in position 4 and methylated amino acid in position 5 [4,83]. Homo-amino acids are also present in cyanopeptolins detected in the strain [81]. They belong to nonproteinogenic amino acids frequently detected in cyanobacterial peptides [50].

The main reasons for a wide interest in anabaenopeptins are their frequent occurrence in various taxonomic groups of cyanobacteria and their biological activity [23,24,40,51,56,77,84]. The promising activity of APs with respect to pharmaceutical application was documented for TAFIa [56]. The compounds inhibit the activity of the enzyme even at a low nM range [56,59]. TAFIa regulates the process of fibrinolysis; therefore, inhibitors of the enzymes are thought to be good candidates for the development of antithrombotic agents.

In our study, the effects of APs on important metabolic enzymes were also explored. The enzymatic assays showed that the isolated APs were inactive against trypsin and chymotrypsin and had weak effects on thrombin, but only at the highest concentration used in the assay. In fact, with the exception of elastase, APs were reported to have mild or no effects on serine proteases such as trypsin, chymotrypsin or thrombin [21,26,40,44,59]. In contrast, APs were found to inhibit CPA, enzymes catalysing the cleavage of carboxyl-terminal peptide bonds in proteins [85]. Deregulation of CPA can lead to cardiovascular disease or cancer [86]. Structure–activity relationship studies revealed the importance of exocyclic amino acid for the potency of APs [9,87]. The presence of hydrophobic amino acids in this position significantly increased their activity compared with peptides with polar residues, such as Lys or Arg. For example, AP G (Tyr+CO[Lys+Ile+Hty+MeHty+Ile]) inhibits CPA with approximately three orders of magnitude lower IC_50_ value (0.002 μM) than AP H (IC_50_ 3.7 μM), which contains Arg instead of Tyr [9,23,87]. However, the activity of the three APs tested in our study was not in line with these findings. The compounds contain hydrophobic amino acids (Leu* or Val) in the exocyclic position, but their effect on CPA was mild (IC_50_ values from 3.5 μM to 21.1 μM). This discrepancy indicates that besides the exocyclic amino acid residue, other parts of APs’ structure also have an effect on the activity.

## 5. Conclusions

As presented in the study, nontargeted chemical analyses may not allow for the detection of compounds produced by cyanobacteria in trace amounts. Therefore, a simultaneous application of genetic and chemical methods is recommended to reveal the full metabolic profile of the organism. Like many other cyanobacteria strains, *Nostoc edaphicum* CCNP1411 produces several structural variants of anabaenopeptins. The structural diversity of the peptides results in their different activities against specific biological targets. This fact raises the question of why cyanobacteria produce several structural variants of anabaenopeptins and what their natural function is.

## Figures and Tables

**Table 1:** *Nostoc* strains that were identified as anabaenopeptins producers.

*Species*	Strain	Place of Isolation	References
*Nostoc* sp.	CENA543	Brazilian saline–alkaline lake, Nhecolândia, Pantanal	[4]
*Nostoc* sp.		Great Hungarian Plain	[5]
*N. punctiforme*	KVS11	*Blassia pusilla* (lichen) Norway	[55]
*N. insulare*	CBT163		[56]
*Nostoc* sp.	CBT599		[56]
*Nostoc* sp.	CENA352	Southeastern Brazilian coastal forest	[7]
*N. punctiforme*	PCC73102	Plant symbiont	[6]
*Nostoc* sp.	ATCC53789	*Macrozamia* sp.	[56]
*N. calcicola*	CB158	Scotland, Arron Island (lichen)	[56]
*Nostoc* sp.	CENA358		[7]

**Table 2:** The organisation of the anabaenopeptin synthetase gene cluster and domain organisation within the proteins encoded by these genes. Domains are abbreviated as follows: A, adenylation; C, condensation; D, docking; E, epimerisation; M, methyltransferase; PCP, peptidyl carrier protein.

Locus_Tag	Gene	Bp	Domain Organisation				
HUN01_12160	*aptA*[fragment 1]				A	PCP	
	*aptA*[fragment 2]	6957		C	A	PCP	E
HUN01_12150	*aptB*	3231		C	A	PCP	
HUN01_12145	*aptC*[fragment 1]			C	A	PCP	
	*aptC*[fragment 2]	7947		C	A	M	PCP
HUN01_12140	*aptD*	4215	D	C	A	PCP	

**Table 3:** Amino acid residues in the substrate-binding pocket of the adenylation domains, encoded by the *apt*ABCD gene cluster, according to GrsA Phe numbering. Amino acids in brackets mark inconsistencies with references [65,72]; position 331 was not taken into account, as in Challis et al. [72], this position was not considered.

	Proposed AA Activated	Residue Position According to GrsA Phe Numbering								
		235	236	239	278	299	301	322	330	331
*aptA*[fragment 1]	Leu/Ile/Val	D	A	F	F	L	G	[A]	T	F
*aptA*[fragment 2]	Lys	D	[T]	E	[Q]	I	G	S	[I]	I
*aptB*	Leu*	D	A	[L]	F	L	G	[A]	V	F
*aptC*[fragment 1]	Phe	D	L	[G]	T	I	G	[C]	V	I
*aptC*[fragment 2]	Asn	D	[A]	T	K	V	G	E	V	G
*aptD*	Phe	D	A	W	T	[V]	A	G	V	C

Leu* stands for Leu or Ile.

**Table 4:** Anabaenopeptin variants identified in *Nostoc edaphicum* CCNP1411.

Name	Calculated MW	[M+H]^+^ *m*/*z*	Amino Acid Sequence						
			1		2	3	4	5	6
AP806Ne	806.43	807.43	Val	CO	Lys	Leu*	Hph	Asn	Phe
AP820Ne	820.45	821.44	Val	CO	Lys	Leu*	Hph	MeAsn	Phe
AP SA6	834.46	835.47	Leu*	CO	Lys	Leu*	Hph	MeAsn	Phe
AP836Ne	836.44	837.50	Val	CO	Lys	Leu*	Hty	MeAsn	Phe

Leu* stands for Leu or Ile.

**Table 5:** NMR spectroscopic data for anabaenopeptin AP820Ne.

Residue	Position	δ_H_ (*J* in Hz)	Residue	Position	δ_H_ (*J* in Hz)
Val	NH 1 2 3 4	6.62 (d, 6.7) 3.85 (m) 1.53 (m) 1.11 (d, 6.7) 1.02 (d, 6.9)	Hph	NH 1 2 3 4/4′ 5/5′ 6	8.82 (d, 3.9) 4.03 (t, 7.7) 2.73, 2.61 1.72 (m) 7.11 (m) 7.32 (d, 7.3) 7.27 (m)
Lys	NH 1 2 3 4 5 ε-NH	7.49 (d, 6.1) 3.53 (m) 1.42 (m) 1.33 (m) 1.42 (m) 2.69 (m) 6.05 (m)	*N*-MeAsn	N-CH_3_1 2 δ-NH_2_	1.81 (s) 5.33 (dd, 9.8, 3.3) 2.41, 2.35 (m) 7.49, 7.03 (m)
Leu*	NH 1 2 3 4 5	7.06 (m) 3.96 (d, 6.6) 1.50 (m) 1.73 (m) 0.95 (m) 0.77 (dd, 6.8, 2.3)	Phe	NH 1 2 3/3′ 4/4′ 5	9.02 (d, 8.9) 4.32 (m) 3.30, 2.77 (m) 7.01 (m) 7.19 (d, 7.6) 7.15 (m)

Leu* stands for Leu or Ile.

**Table 6:** The activities of the peptides were assessed in serine proteases (chymotrypsin (CHY), trypsin (TRY), elastase (E), and thrombin (Thr)) and carboxypeptidase A (CPA) inhibition assays: –, not active (inhibition below 10%); *, low activity (inhibition between 10–30%); **, medium activity (inhibition between 31–70%).

Name	Enzyme Inhibition (IC_50_ [µM])				
	TRY	CHY	E	CPA	Thr
AP SA6	*–*	*–*	5.5	4.5	*
AP820Ne	*–*	*–*	22.7	3.5	**
AP806Ne	*–*	*–*	*–*	21.1	*

## Data Availability

Not applicable.

## Supplementary Materials

The following supporting information can be downloaded at: https://www.mdpi.com/article/10.3390/ijerph191912346/s1, Figure S1: schematic alignment of genes coding for anabaenopeptin synthetase from *N. edaphicum* CCNP1411 and three related *apt* regions encoding synthetases from *Nodularia spumigena* CCY9414 (CP007203.2), *Anabaena* sp. 90 (GU174493.1) and *Nostoc punctiforme* PCC 73102 (NC_010628.1). The grey bar in the upper right corner shows the identity percentage associated with the colour of the bars connecting homologous regions. Red colour represents genes of core anabaenopeptin biosynthetic gene cluster, burgundy colour represents putative *hphA* gene and dark grey represents the *aptE* gene coding for an ATP binding cassette transporter. NRPS from *Nostoc punctiforme* PCC 73102 is portrayed in shades of grey, as it does not resemble an *apt* biosynthetic gene cluster. Schematic alignment of genes was visualised by EasyFig programme (http://mjsull.github.io/Easyfig/files.html, accessed on 23 August 2022), Table S1: anabaenopeptin variants produced by genus *Nostoc*, Table S2: conditions and solvents used in the enzyme inhibition assays, Figure S2: MRM chromatograms of anabaenopeptins produced by *N. edaphicum* CCNP1411, Figure S3: structure and enhanced product ion mass spectrum of the anabaenopeptin AP806Ne [Lys–Leu*–Hph–Asn–Phe]CO–Val with precursor ion [M+H]^+^ at *m*/*z* 807. The mass signals were assigned to the following fragments: 807 [M+H]^+^, 790 [M+2H–H_2_O]^+^, 789 [M+H–H_2_O]^+^, 779 [M+H–CO]^+^, 762 [M+2H–CO–H_2_O]^+^, 744 [M+2H–CO–2H_2_O]^+^, 708 [M+H–Val]^+^, 694 [M+H–Leu*]^+^, 690 [M+H–Val–H_2_O]^+^, 664 [M+H–(Val–CO)]^+^, 646 [M+H–Hph]^+^, 629 [M+2H–Hph–H_2_O]^+^, 619 [M+2H–Hph–CO]^+^, 601 [M+2H–Hph–CO–H_2_O]^+^, 536 [M+H–(Lys–CO–Val)]^+^, 533 [M+H–(Leu*+Hph)]^+^, 518 [M+H–(Lys–CO–Val)-H_2_O]^+^, 515 [M+H–(Leu*–Hph)–H_2_O]^+^, 506 [M+2H–(Leu*+Hph) –CO]^+^, 504 [M+H–(Asn+Phe)–CO]^+^, 434 [M+H–(Hph+Leu*)–Val]^+^, 420 [M+2H–(Leu*+Hph+Asn)]^+^, 402 [M+2H–(Leu*+Hph+Asn)–H_2_O]^+^, 390 [Asn+Phe+Lys+H]^+^, 362 [Asn+Phe+Lys+H–CO]^+^, 343 [Leu*+Phe+Lys+H–CO-H_2_O]^+^, 276 [Hph+Asn+H]^+^, 262 [Phe+Asn+H]^+^, 248 [Phe+Lys+H–CO]^+^, 234 [Phe+Asn+H–CO]^+^, 173 [Lys+CO+NH_2_+H]^+^, 134 Hph immonium ion, 129 [Lys+2H]^+^, 120 Phe immonium ion, 86 Leu* immonium ion, 84 Lys-derived ions^+^ and 72 Val immonium ion, Figure S4: structure and enhanced product ion mass spectrum of the anabaenopeptin AP SA6 [Lys–Leu*–Hph–MeAsn–Phe]CO–Leu* with precursor ion [M+H]^+^ at *m*/*z* 835. The mass signals were assigned to the following fragments: 835 [M+H]^+^, 817 [M+H–H_2_O]^+^, 807 [M+H–CO]^+^, 800 [M+H–NH_3_–H_2_O]^+^, 789 [M+H–CO–H_2_O]^+^, 772 [M+H–CO–H_2_O–NH_3_]^+^, 722 [M+H–Leu*]^+^, 707 [M+H–MeAsn]^+^, 704 [M+H–Leu*–H_2_O]^+^, 678 [M+H–(CO+Leu*)]^+^, 674 [M+H–Hph]^+^, 660 [M+H–(CO+Leu*)–H_2_O]^+^, 656 [M+H–Hph–H_2_O]^+^, 561 [M+H–(Leu*+Hph)]^+^, 550 [M+H–(Lys+CO+Leu*)]^+^, 533 [M+H–(Leu*+Hph)–CO]^+^, 448 [M+H–Leu*–(Hph+Leu*)]^+^, 403 [Leu*+Hph+MeAsp+H]^+^, 276 [Phe+MeAsn+H]^+^, 248 [Phe+MeAsn+H–CO]^+^, 173 [Lys+CO+NH_2_+H]^+^, 134 Hph immonium ion, 129 [Lys+2H]^+^, 84 Lys-derived ions^+^, 120 Phe immonium ion, 101 MeAsn immonium and 86 Leu* immonium ion, Figure S5: structure and enhanced product ion mass spectrum of the anabaenopeptin AP836Ne [Lys–Leu*–Hty–MeAsn–Phe]CO–Leu* with precursor ion [M+H]^+^ at *m*/*z* 837. The mass signals were assigned to the following fragments: 837 [M+H]^+^, 819 [M+H–H_2_O]^+^, 809 [M+H–CO]^+^, 802 [M+H–NH_3_–H_2_O]^+^, 791 [M+H–CO–H_2_O]^+^, 738 [M+H–Val]^+^, 724 [M+H–Leu*]^+^, 720 [M+H–Val-H_2_O]^+^, 709 [M+H–MeAsn]^+^, 694 [M+H–(CO+Val)]^+^, 660 [M+H–Hty]^+^, 566 [M+H–(Lys+CO+Val)]^+^, 547 [M+H–(Leu*+Hty)]^+^, 519 [M+H–(Leu*+Hty)–CO]^+^, 448 [M+H–Val–(Hty+Leu*)]^+^, 419 [M+H–(Leu*+Hty+MeAsn)]^+^, 388 [Leu*+Lys+Phe+H]^+^, 276 [Phe+MeAsn+H]^+^, 248 [Phe+MeAsn+H–CO]^+^, 173 [Lys+CO+NH_2_+2H]^+^, 150 Hty immonium ion, 129 [Lys+2H]^+^, 84 Lys-derived ions^+^, 120 Phe immonium ion, 101 MeAsn immonium and 72 Val immonium ion, Figure S6: ^1^H NMR spectrum of anabaenopeptin AP820Ne in DMSO-d_6_, Figure S7: COSY spectrum of anabaenopeptin AP820Ne in DMSO-d_6_, Figure S8: TOCSY spectrum of anabaenopeptin AP820Ne in DMSO-d_6_, Figure S9: ROESY spectrum of anabaenopeptin AP820Ne in DMSO-d_6_. Reference [88] is cited in the Supplementary Materials.
