# Supplementary material for: Anabaenopeptins from Nostoc edaphicum CCNP1411
Source: Int J Environ Res Public Health. 2022 Sep 28;19(19):12346. doi: 10.3390/ijerph191912346 (PMC9564503; doi:10.3390/ijerph191912346)
Supplement: Supplementary file 1 [file ijerph-19-12346-s001.zip › ijerph-1924476-supplementary.pdf]

Supplementary Material: Anabaenopeptins produced by *Nostoc edaphicum* CCNP1411

Robert Konkel[1], Michał Grabski[2], Marta Cegłowska[3], Ewa Wieczerzak[4], Grzegorz Węgrzyn[2], Hanna Mazur-Marzec[1*]

1. Division of Marine Biotechnology, Institute of Oceanography, University of Gdańsk, M. J. Piłsudskiego 46, PL-81378 Gdynia, Poland
2. Department of Molecular Biology, University of Gdansk, Wita Stwosza 59, 80-308 Gdansk, Poland
3. Institute of Oceanology, Polish Academy of Sciences, Powstańców Warszawy 55, PL-81712 Sopot, Poland
4. Department of Biomedical Chemistry, Faculty of Chemistry, University of Gdańsk, Wita Stwosza 63, PL-80308 Gdańsk, Poland

*Correspondence: hanna.mazur-marzec@ug.edu.pl

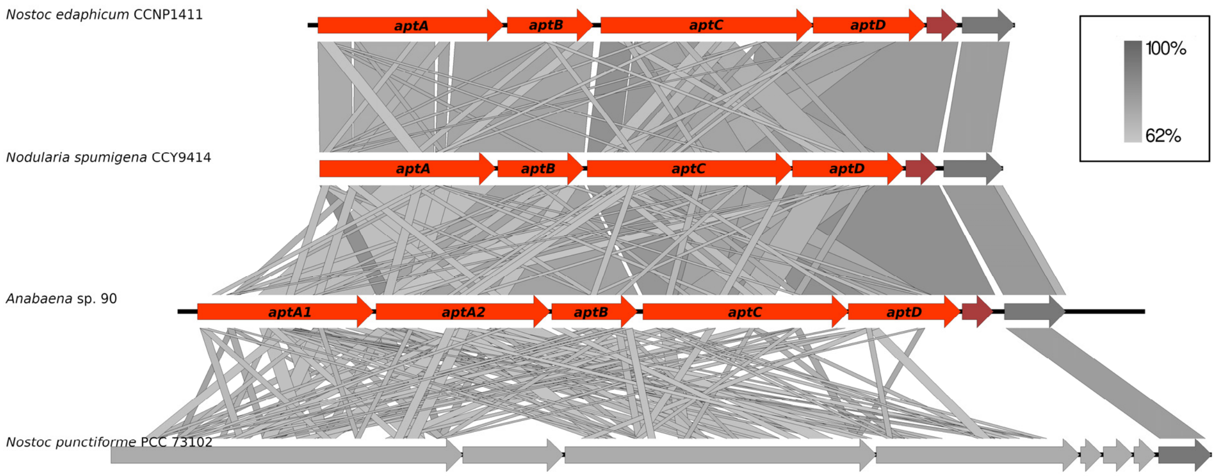

Figure S1. Schematic alignment of genes coding for anabaenopeptin synthetase from *N. edaphicum* CCNP1411 and three related *apt* regions encoding synthetases from *Nodularia spumigena* CCY9414 (CP007203.2), *Anabaena* sp. 90 (GU174493.1), and *Nostoc punctiforme* PCC 73102 (NC_010628.1). The Gray bar in the upper right corner shows the identity percentage associated with the colour of the bars connecting homologous regions. Red colour represents genes of core anabaenopeptin biosynthetic gene cluster, burgundy colour represents putative *hphA* gene and dark grey represents the *aptE* gene, coding for an ATP binding cassette transporter. NRPS from *Nostoc punctiforme* PCC 73102 is portrayed in shades of grey as it does not resemble an *apt* biosynthetic gene cluster. Schematic alignment of genes was visualized by EasyFig program (http://mjsull.github.io/Easyfig/files.html, accessed on 23 August 2022).

Table S1. Anabaenopeptin variants produced by the genus *Nostoc*.

Name	m/z [M+H]$^+$	Amino Acid Sequence							Ref
Nostamide C	778.45	Ile	CO	Lys	Val	Hph	MeAla	Phe	[4]
Nostamide D	778.45	Val	CO	Lys	Ile	Hph	MeAla	Phe	[4]
Nostamide E	778.45	Ile	CO	Lys	Ile	Hph	Ala	Phe	[4]
Schizopeptin 791	792	Ile	CO	Lys	Ile	Hph	MeAla	Phe	[4,5,44]
Nostamide B	806.46	Ile	CO	Lys	Ile	Hph	MeAla	Hph	[4]
AP 807	808	Ile	CO	Lys	Ile	Hty	MeAla	Phe	[4,5]
AP KVJ811	812.43	Tyr	CO	Lys	Ile	Hph	MeGly	Hph	[55]
AP 827B	828.38	Phe	CO	Lys	Val	Hph	Ala	Hty	[5]
AP KVJ827	828.43	Tyr	CO	Lys	Val	Hph	MeGly	Hph	[55]
	829.0								[76]
AP SA5	835.00	Ile	CO	Lys	Val	PNV	Asn	Phe	[56]
AP SA6	835.00	Ile	CO	Lys	Ile	Hphe	Asn	Phe	[56]
AP 834	835.31	Ile/Leu	CO	Lys	Val	Hph	MeAsn	Phe	[5]
Nostamide A	842.0	Phe	CO	Lys	Ile	Hph	MeGly	Hty	[6]
AP 841B	842.44	Phe	CO	Lys	Val	Hph	MeAla	Hty	[5,7]
AP KVJ841	842.45	Phe	CO	Lys	Val	Hph	MeGly	Hph	[55]
AP SA12	843.96	Phe	CO	Lys	Val	Hty	MeGly	Hty	[56]
	845.2								[77]
AP 848	849.34	Ile	CO	Lys	Val	MeHph	MeAsn	Hph	[5]
AP SA7	849.49	Ile	CO	Lys	Ile	PNV	Asn	Phe	[56]
	842.9								[77]
AP 855 A	856.46	Phe	CO	Lys	Ile	Hph	MeAla	Hty	[5,8]
AP NZ857	858	Phe	CO	Lys	Ile	Hty	MeGly	Hty	[6,31]
AP 857 A	858.44	Phe	CO	Lys	Val	Hty	MeAla	Hty	[7]
	859.1								[76]
AP 862 A	863.26	Ile/Leu	CO	Lys	Val	EtHph	MeAsn	Hph	[5]
AP SA8	863.50	Ile	CO	Lys	Ile	PNL	Asn	Phe	[56]
AP SA4	864.04	Lys	CO	Lys	Ile	PNV	Asn	Phe	[56]
AP 864	865.3	Ile/Leu	CO	Lys	Val	EtHph	MeAsn	Hty	[5]
AP 869	870.16	Tyr	CO	Lys	Ile/Leu	Hph	MeAla	MeHph	[5]
AP 871	872.45	Phe	CO	Lys	Ile	Hty	MeAla	Hty	[7]

AP SA9	876.43	Phe	CO	Lys	Ile	Cl-Hty	MeGly	Hphe	[56]
AP 876	877.16	Ile/Leu	CO	Lys	Ile/Leu	EtHph	MeAsn	Hph	[5]
AP SA10	878.38	Phe	CO	Lys	Ile	Hty	MeGly	Cl-Hty	[56]
AP 882	883.47	Phe	CO	Lys	Ile	MeHph	MeAsn	Phe	[7]
AP SA1	892.05	Arg	CO	Lys	Ile	PNV	Asn	Phe	[56]
AP SA11	892.43	Phe	CO	Lys	Ile	Hty	MeGly	Cl-Hty	[56]
AP 896	897.49	Phe	CO	Lys	Ile	EtHph	MeAsn	Phe	[7]
AP KB899	900.31	Tyr	CO	Lys	Val	Hph	MeHty	Ile	[5,88]
AP MM913	914.50	Tyr	CO	Lys	Ile	Hph	MeHty	Ile	[5,40]
AP 915	916.48	Tyr	CO	Lys	Val	Hty	MeHty	Ile	[5,26]
AP G	930.50	Tyr	CO	Lys	Ile	Hty	MeHty	Ile	[5,23]

Table S2. Conditions and solvents used in the enzyme inhibition assays.

Enzyme	Substrate	Inhibitor	Preincubation	Reaction time (min)	Wavelength (nm)
Carboxypeptidase-A (Sigma-Aldrich; St. Louis, MO, USA) 1.6 µM	*N*-(4-methoxy-phenyl-azoformyl)-Phe-OH (GLPBIO Technology LLC; Montclair, CA, USA) 0.2 mg mL^{-1}	Carboxypeptidase inhibitor from potato tuber (Sigma-Aldrich) 2.5–150 µg mL^{-1}	5 min; 25 °C	10	350
Chymotrypsin (Sigma-Aldrich) 0.1 mg mL^{-1}	Suc-Gly-Gly-p-nitroanilide (Sigma-Aldrich) 2 mM	Aprotinine (Sigma-Aldrich) 1.5–200 µg mL^{-1}	5 min; 25 °C	20	405
Elastase (Sigma-Aldrich) 75 µg mL^{-1}	*N*-succinyl-Ala-Ala-Ala-*p*-nitroanilide (Sigma-Aldrich) 2 mM	Elastatinal (Sigma-Aldrich) 1.5–250 µg mL^{-1}	20 min; 30 °C	10	405
Thrombin (Sigma-Aldrich) 0.5 mg mL^{-1}	*N*-*p*-tosyl-Gly-Pro-Lys-*p*-nitroanilide acetate salt (Sigma-Aldrich) 0.5 mg mL^{-1}	AEBSF (Sigma-Aldrich) 60–5000 µg mL^{-1}	10 min; 25 °C	10	405
Trypsin (Sigma-Aldrich) 0.1 mg mL^{-1}	*N*-*α*-benzoyl-DL-arginine-*p*-nitroanilide hydrochloride (BAPNA; Sigma-Aldrich) 2 mM	Aprotinine (Sigma-Aldrich) 2.5–125 µg mL^{-1}	15 min; 25 °C	20	405

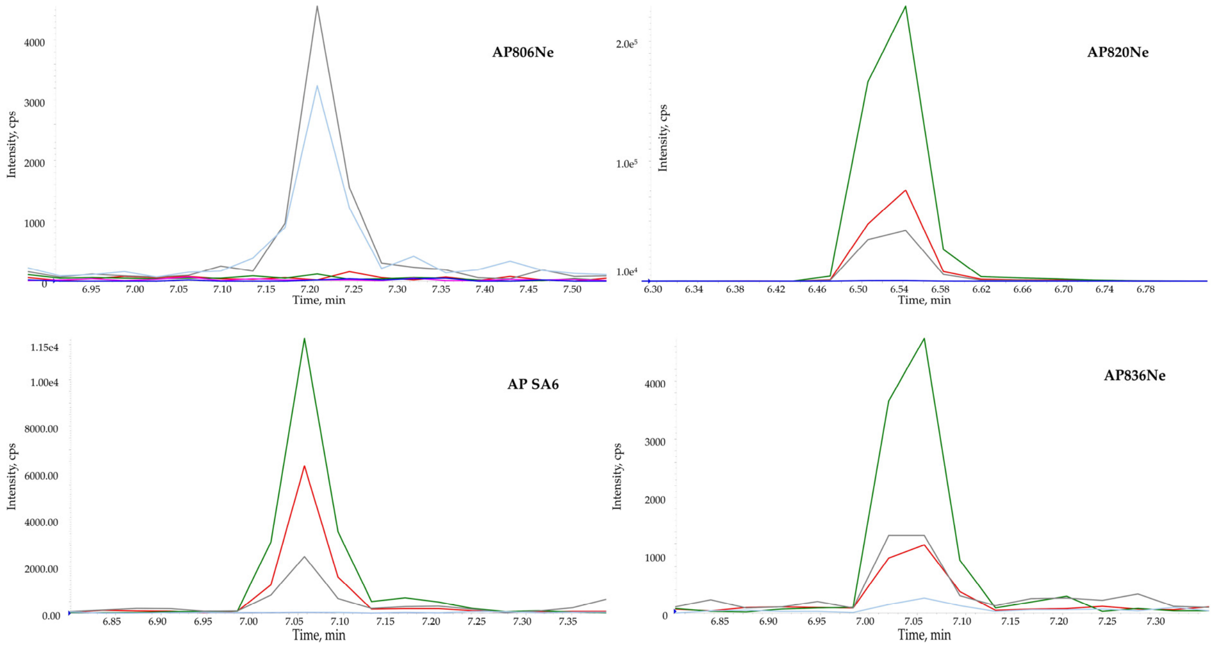

Figure S2. MRM chromatograms of anabaenopeptins produced by *N. edaphicum* CCNP1411.

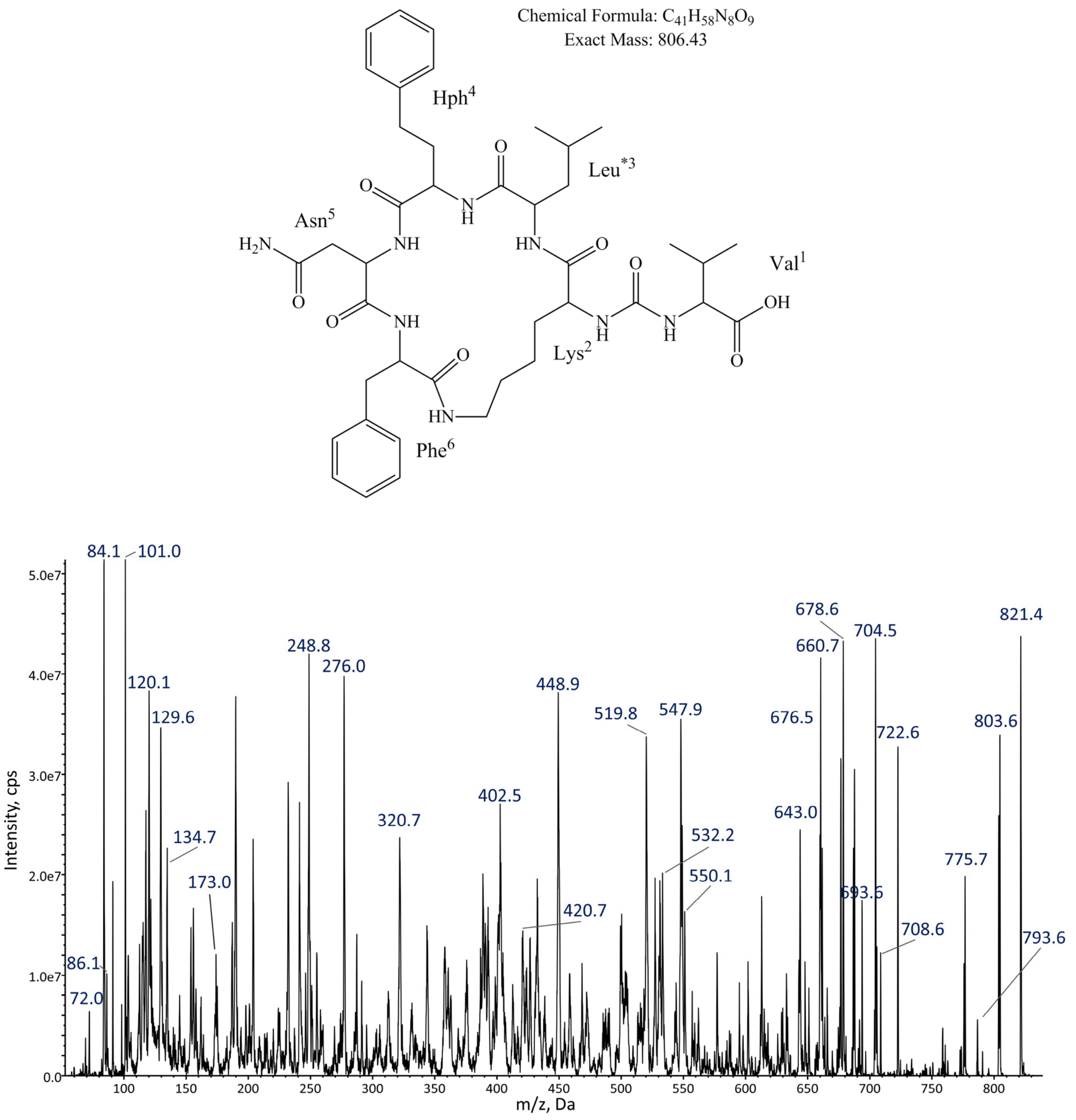

Figure S3. Structure and enhanced product ion mass spectrum of the anabaenopeptin AP806Ne [Lys–Leu*–Hph–Asn–Phe]CO–Val with precursor ion [M+H]$^+$ at *m/z* 807. The mass signals were assigned to the following fragments: 807 [M+H]$^+$, 790 [M+2H–H$_2$O]$^+$, 789 [M+H–H$_2$O]$^+$, 779 [M+H–CO]$^+$, 762 [M+2H–CO–H$_2$O]$^+$, 744 [M+2H–CO–2H$_2$O]$^+$, 708 [M+H–Val]$^+$, 694 [M+H–Leu*]$^+$, 690 [M+H–Val–H$_2$O]$^+$; 664 [M+H–(Val–CO)]$^+$, 646 [M+H–Hph]$^+$, 629 [M+2H–Hph–H$_2$O]$^+$, 619 [M+2H–Hph–CO]$^+$, 601 [M+2H–Hph–CO–H$_2$O]$^+$, 536 [M+H–(Lys–CO–Val)]$^+$, 533 [M+H–(Leu*+Hph)]$^+$, 518 [M+H–(Lys–CO–Val)-H$_2$O]$^+$, 515 [M+H–(Leu*–Hph)–H$_2$O]$^+$, 506 [M+2H–(Leu*+Hph) –CO]$^+$, 504 [M+H–(Asn+Phe)–CO]$^+$, 434 [M+H–(Hph+Leu*)–Val]$^+$, 420 [M+2H–(Leu*+Hph+Asn)]$^+$, 402 [M+2H–(Leu*+Hph+Asn)–H$_2$O]$^+$, 390 [Asn+Phe+Lys+H]$^+$, 362 [Asn+Phe+Lys+H–CO]$^+$, 343 [Leu*+Phe+Lys+H–CO-H$_2$O]$^+$, 276 [Hph+Asn+H]$^+$, 262 [Phe+Asn+H]$^+$, 248 [Phe+Lys+H–CO]$^+$, 234 [Phe+Asn+H–CO]$^+$, 173 [Lys+CO+NH$_2$+H]$^+$, 134 Hph immonium ion, 129 [Lys+2H]$^+$, 120 Phe immonium ion, 86 Leu* immonium ion, 84 Lys-derived ions$^+$, 72 Val immonium ion.

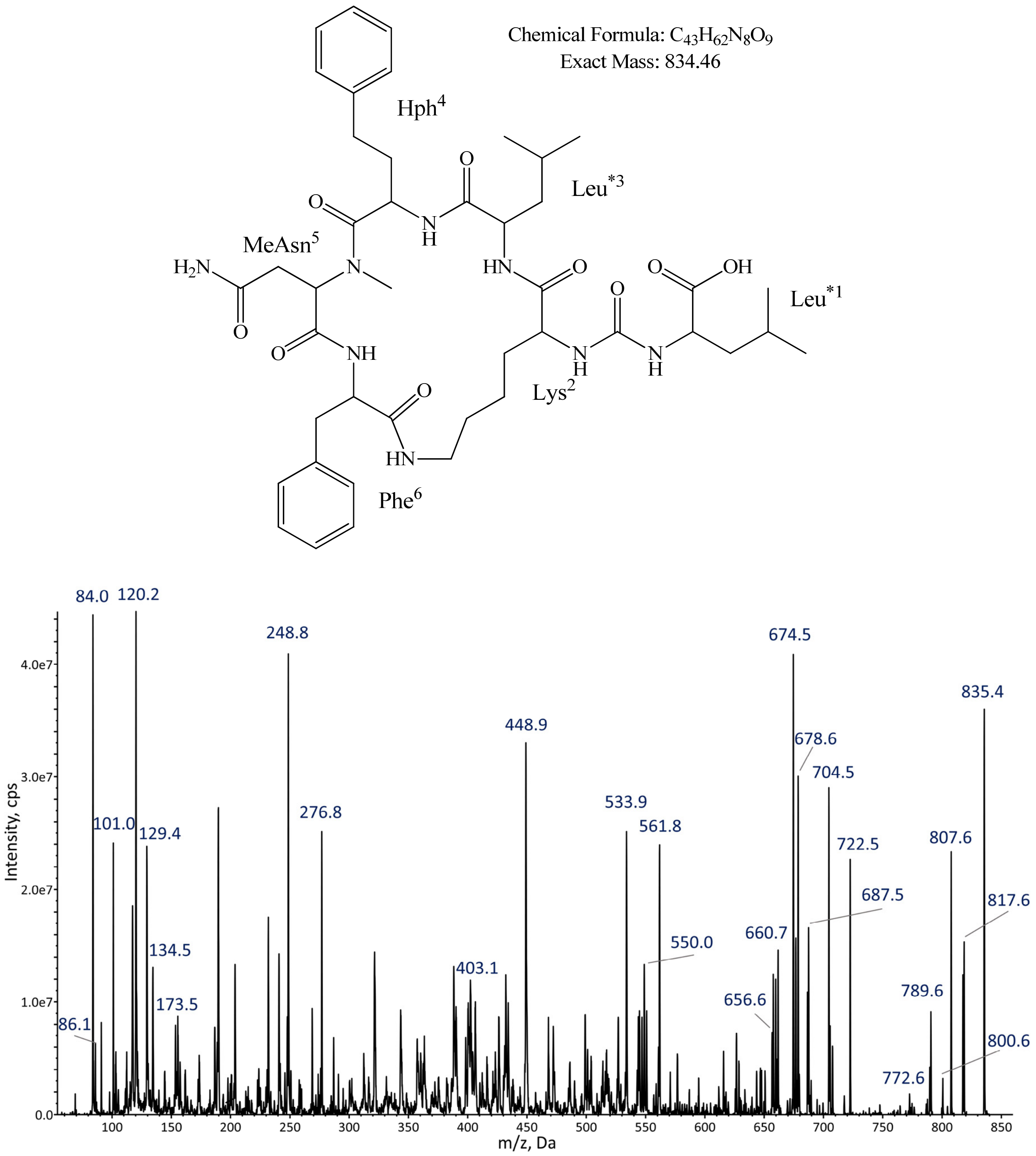

Figure S4. Structure and enhanced product ion mass spectrum of the anabaenopeptin AP SA6 [Lys–Leu*–Hph–MeAsn–Phe]CO–Leu* with precursor ion $[M+H]^+$ at *m/z* 835. The mass signals were assigned to the following fragments: 835 $[M+H]^+$, 817 $[M+H–H_2O]^+$, 807 $[M+H–CO]^+$, 800 $[M+H–NH_3–H_2O]^+$, 789 $[M+H–CO–H_2O]^+$, 772 $[M+H–CO–H_2O–NH_3]^+$, 722 $[M+H–Leu^*]^+$, 707 $[M+H–MeAsn]^+$, 704 $[M+H–Leu^*–H_2O]^+$, 678 $[M+H–(CO+Leu^*)]^+$, 674 $[M+H–Hph]^+$, 660 $[M+H–(CO+Leu^*)–H_2O]^+$, 656 $[M+H–Hph–H_2O]^+$, 561 $[M+H–(Leu^*+Hph)]^+$, 550 $[M+H–(Lys+CO+Leu^*)]^+$, 533 $[M+H–(Leu^*+Hph)–CO]^+$, 448 $[M+H–Leu^*–(Hph+Leu^*)]^+$, 403 $[Leu^*+Hph+MeAsp+H]^+$, 276 $[Phe+MeAsn+H]^+$, 248 $[Phe+MeAsn+H–CO]^+$, 173 $[Lys+CO+NH_2+H]^+$, 134 Hph immonium ion, 129 $[Lys+2H]^+$, 84 Lys-derived ions$^+$, 120 Phe immonium ion, 101 MeAsn immonium, 86 Leu* immonium ion.

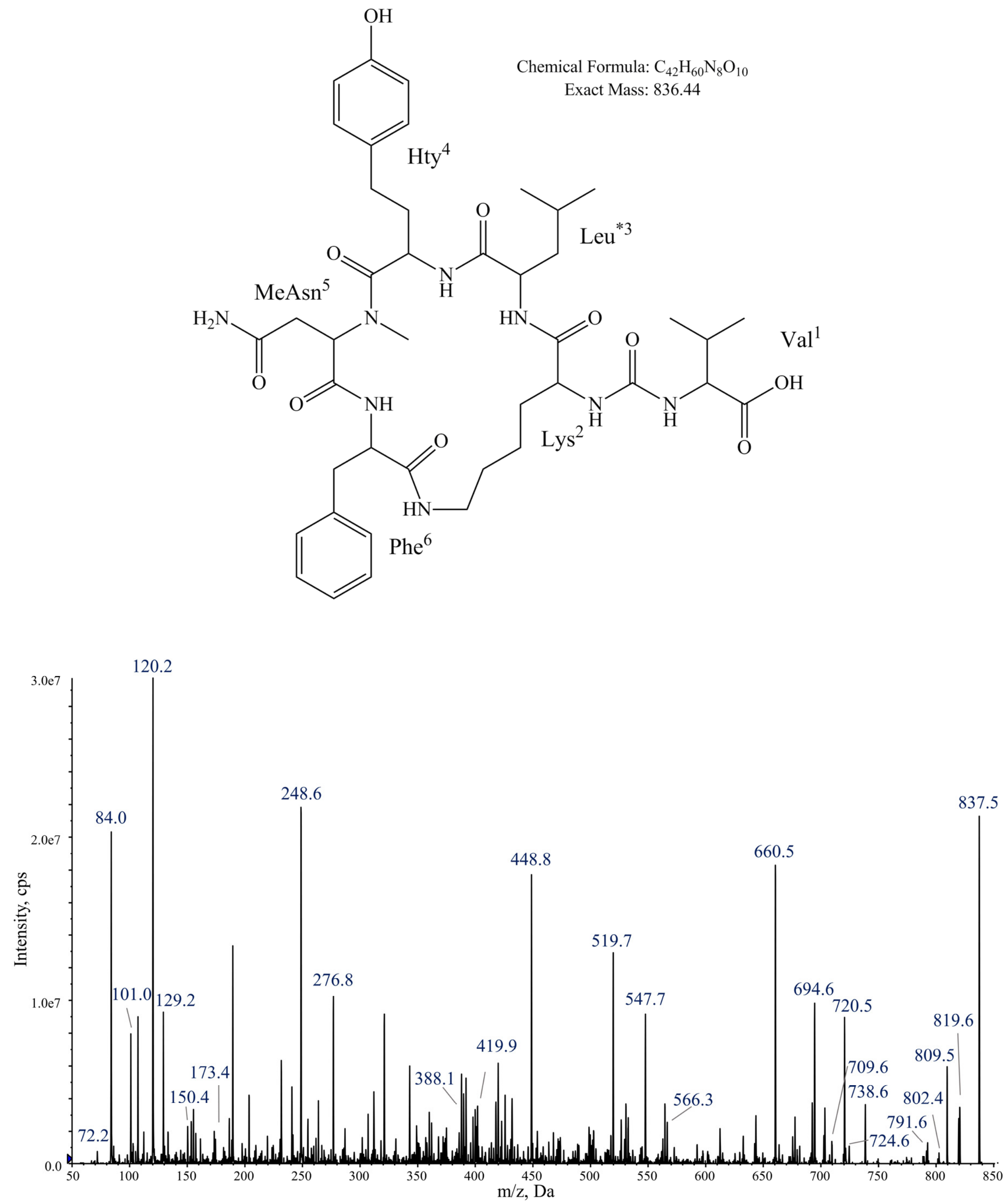

Figure S5. Structure and enhanced product ion mass spectrum of the anabaenopeptin AP836Ne [Lys–Leu*–Hty–MeAsn–Phe]CO–Leu* with precursor ion $[M+H]^+$ at *m/z* 837. The mass signals were assigned to the following fragments: 837 $[M+H]^+$, 819 $[M+H–H_2O]^+$, 809 $[M+H–CO]^+$, 802 $[M+H–NH_3–H_2O]^+$, 791 $[M+H–CO–H_2O]^+$, 738 $[M+H–Val]^+$, 724 $[M+H–Leu^*]^+$, 720 $[M+H–Val-H_2O]^+$, 709 $[M+H–MeAsn]^+$, 694 $[M+H–(CO+Val)]^+$, 660 $[M+H–Hty]^+$, 566 $[M+H–(Lys+CO+Val)]^+$, 547 $[M+H–(Leu^*+Hty)]^+$, 519 $[M+H–(Leu^*+Hty)–CO]^+$, 448 $[M+H–Val–(Hty+Leu^*)]^+$, 419 $[M+H–(Leu^*+Hty+MeAsn)]^+$, 388 $[Leu^*+Lys+Phe+H]^+$, 276 $[Phe+MeAsn+H]^+$, 248 $[Phe+MeAsn+H–CO]^+$, 173 $[Lys+CO+NH_2+2H]^+$, 150 Hty immonium ion, 129 $[Lys+2H]^+$, 84 Lys-derived ions$^+$, 120 Phe immonium ion, 101 MeAsn immonium, 72 Val immonium ion.

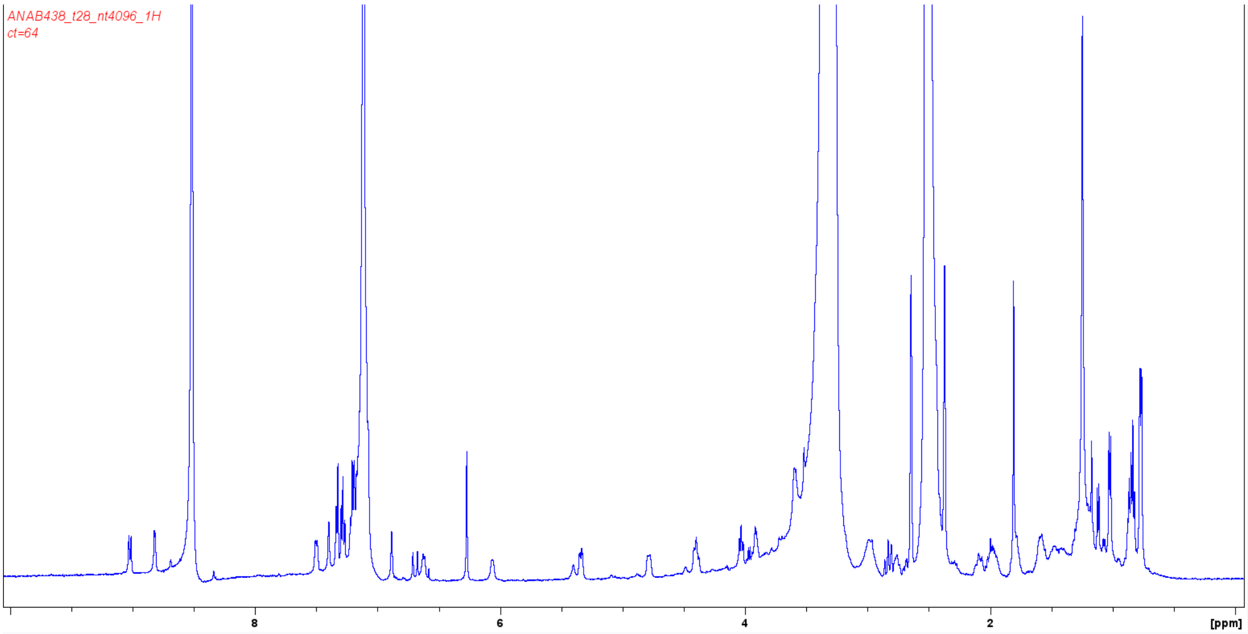

Figure S6. ^{1}H NMR spectrum of anabaenopeptin AP820Ne in DMSO-d_6.

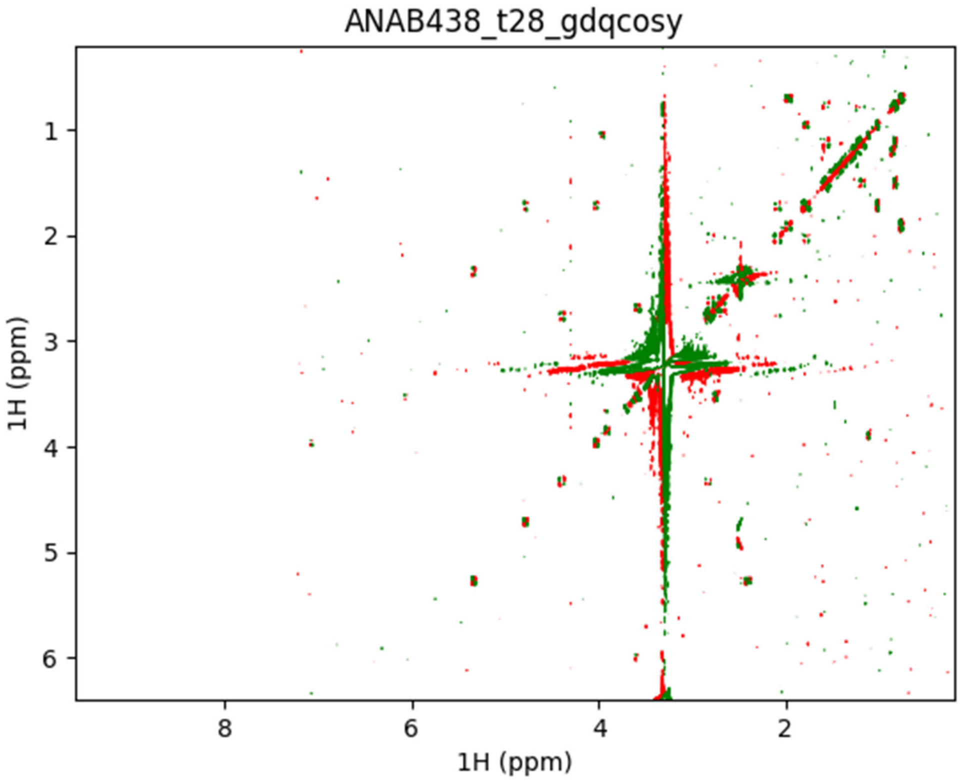

Figure S7. COSY spectrum of anabaenopeptin AP820Ne in DMSO-d_6.

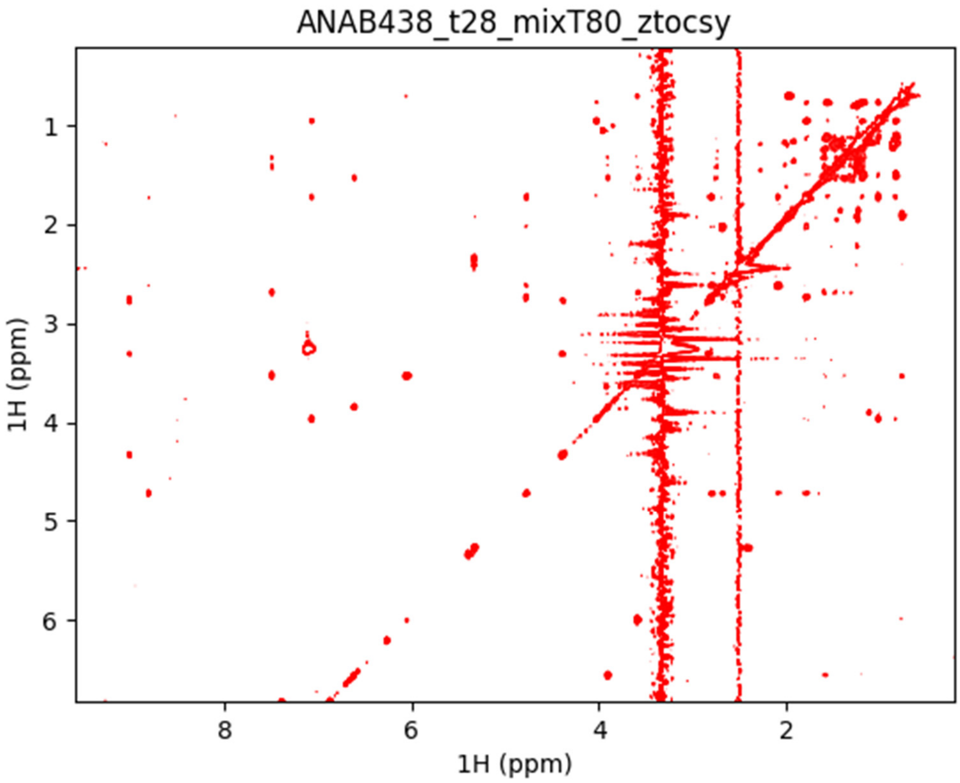

Figure S8. TOCSY spectrum of anabaenopeptin AP820Ne in DMSO-d_6.

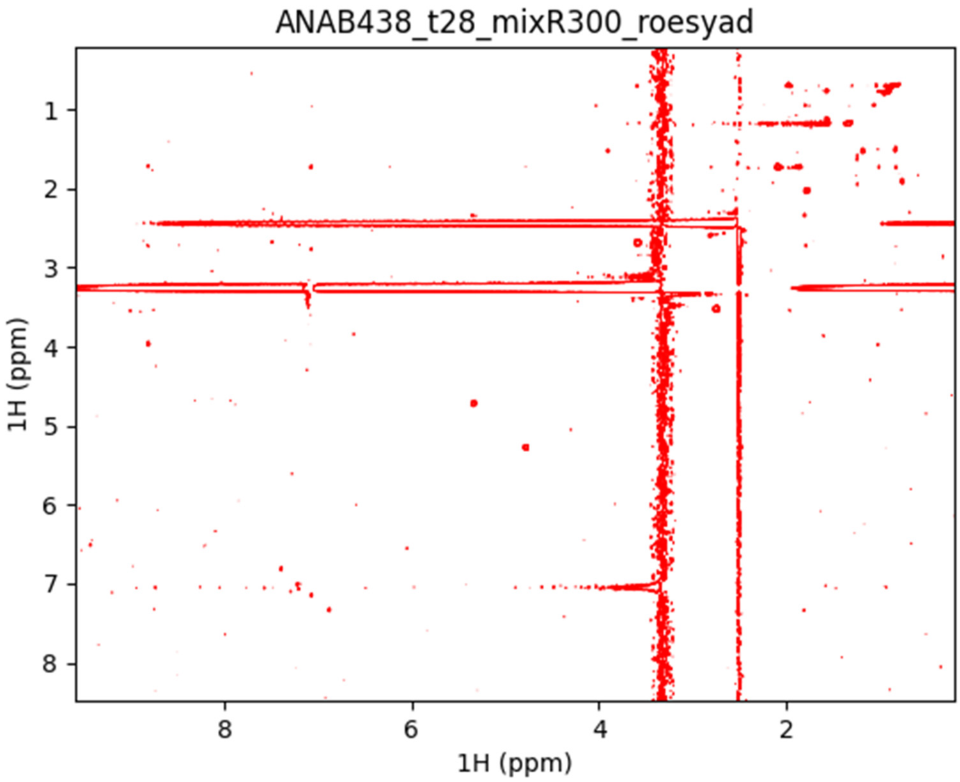

Figure S9. ROESY spectrum of anabaenopeptin AP820Ne in DMSO-d_6.
